# Exploring Therapeutic Challenges in Patients with HER2-Positive Breast Cancer—A Single-Center Experience

**DOI:** 10.3390/life14081025

**Published:** 2024-08-18

**Authors:** Ramona Coca, Andrei Moisin, Rafaela Coca, Atasie Diter, Mihaela Racheriu, Denisa Tanasescu, Carmen Popa, Maria-Emilia Cerghedean-Florea, Adrian Boicean, Ciprian Tanasescu

**Affiliations:** 1 Clinical Medical Department, Faculty of General Medicine, “Lucian Blaga” University of Sibiu, Str. Lucian Blaga nr. 2A, 550169 Sibiu, Romania; ramona.coca@ulbsibiu.ro (R.C.); rafaela.coca@ulbsibiu.ro (R.C.); diter.atasie@ulbsibiu.ro (A.D.); adrian.boicean@ulbsibiu.ro (A.B.); 2 Department of Oncology, Sibiu County Emergency Clinical Hospital, B-dul Corneliu Coposu nr. 2-4, 550245 Sibiu, Romania; 3 Surgical Clinical Department, Faculty of General Medicine, “Lucian Blaga” University of Sibiu, Str. Lucian Blaga nr. 2A, 550169 Sibiu, Romania; mihaela.racheriu@ulbsibiu.ro (M.R.); mariaemilia.florea@ulbsibiu.ro (M.-E.C.-F.); ciprian.tanasescu@ulbsibiu.ro (C.T.); 4 Department of Surgery, Sibiu County Emergency Clinical Hospital, B-dul Corneliu Coposu nr. 2-4, 550245 Sibiu, Romania; 5 Department of Radiology and Medical Imaging, Sibiu County Emergency Clinical Hospital, B-dul Corneliu Coposu nr. 2-4, 550245 Sibiu, Romania; carmen.0306@yahoo.com; 6 Department of Nursing and Dentistry, Faculty of General Medicine, “Lucian Blaga” University of Sibiu, Str. Lucian Blaga nr. 2A, 550169 Sibiu, Romania; denisa.tanasescu@ulbsibiu.ro

**Keywords:** HER2, ERBB2 protein, breast cancer, tyrosine kinase receptors

## Abstract

Breast cancer is one of the most common forms of neoplasia worldwide. The purpose of our observational study was to evaluate the status of HER2 overexpression among new cases of breast neoplasia with an impact on the natural history of breast cancer disease and therapeutic personalization according to staging. This study included 45 breast cancer patients which have an overexpression of HER2 through the mutation of the EGFR-ERBB2 receptor. Immunohistochemical staining was performed on sections of formalin-fixed paraffin-embedded breast tissue. The patients were evaluated demographically and therapeutically in all stages. The post-surgical histopathological examination revealed complete pathological responses in 19 patients and pathological responses with residual disease either at the tumor level or lymphatic or both variants in a percentage of 44% (15 cases). The disease-free interval (DFI) under anti-HER2 therapy was recorded in 41 patients, representing 91% of the study group. Anti-HER2 therapy in any therapeutic stage has shown increased efficiency in blocking these tyrosine kinase receptors, evidenced by the high percentage of complete pathological responses, as well as the considerable percentage (47%) of complete remissions and stationary disease, in relation to the HER2-positive patient group.

## 1. Introduction

The increased incidence of breast cancer and the presence of various malignant phenotypes require the search for an optimal, systematic strategy for the diagnosis and treatment of this neoplasia [1,2]. The current oncological therapeutic concept is to customize the therapy in order to increase the disease-free interval (for early stages) and progression-free survival (in metastatic disease) as well as the overall survival [2,3]. The decision making process in breast cancer management should include a detailed discussion with the patient regarding the need for essential genetic testing [4,5]. Multigenic test panels have a positive impact on establishing an individualized therapeutic approach, both for patients and their descendants (knowledge of and reduction in neoplasia risk) [6,7]. Limited testing of BReast CAncer gene 1 (BRCA 1) and BReast CAncer gene 2 (BRCA 2) mutations may lead to the omission of other genetic changes that radically influence the therapeutic approach, with negative implications for increasing overall survival [7,8]. BRCA 1/2 germ mutation testing as well as multigenic panels do not have settlement systems (full or co-payment) through the National Health Insurance House [9]. The price of these genetic determinations is high, which is why, due to the current socioeconomic conditions, only a small percentage of patients can afford BRCA testing [9,10,11,12].

In HER2-positive breast cancer, the overexpression of HER2 is identified in a percentage range of 15–20% of all cases of invasive breast carcinomas and in approximately 10% of breast cancers with positive estrogenic receptors [13,14]. In the neoadjuvant setting, the standard of care is represented by the double anti-HER2 blockade: Pertuzumab + Trastuzumab associated with chemotherapy with Taxanes, according to the guidelines from the ESMO (European Society of Medical Oncology) and NCCN (National Coperhensive Cancer Network) [15,16].

The addition of anti-HER2 therapy to the neoadjuvant management of early-stage HER2-positive breast cancer has yielded substantial clinical benefits, dramatically improving patient outcomes in HER2-positive (HER2+) early breast cancer, as recently proven by the 2021 EBCTCG (Early Breast Cancer Trialists’ Collaborative Group) meta-analysis of Trastuzumab in HER2-positive early breast cancer [17,18]. Although pCR (pathological complete response) patients have a very favorable outcome with adjuvant trastuzumab (+/− pertuzumab), adjuvant T-DM1 (Trastuzumab–Emtansine) offers an important escalation strategy in non-pCR patients in the adjuvant setting [17,18].

For recurrent metastatic disease, anti-HER2 therapy includes several treatment lines, depending on the progression of the disease [19,20]. The pretherapeutic diagnostic evaluation of relapsed metastatic breast cancer includes the revision of the HER2 protein positivity (tumor genetic instability) by a biopsy of metastatic lesions and the identification of PIK3CA genetic mutations as well as the PD-L1 immune receptor [21,22]. Immunotherapy has recently been reimbursed in Romania, in triple negative metastatic breast cancer [21,22].

**Figure 1:**
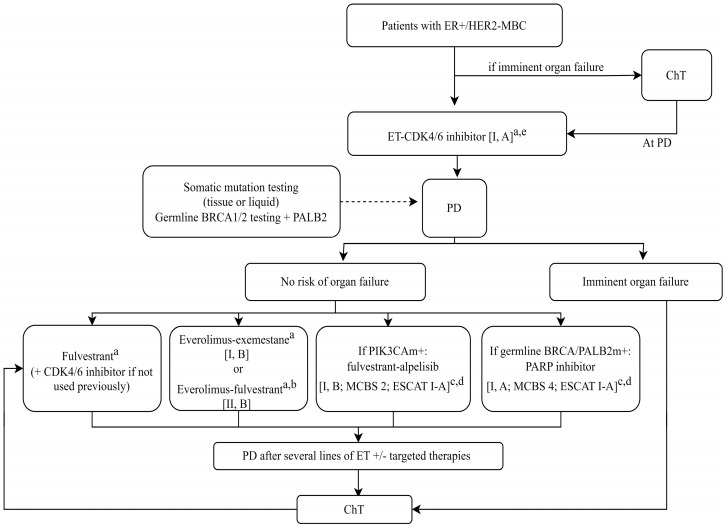
Treatment of metastatic breast cancer with positive estrogenic receptors, HER2−negative, but with BRCA 1 and BRCA2 mutations, +/− MUTATIONS of PIK3CA [23]. Figure legend: CDK4/6, cyclin−dependent kinase 4 and 6; ChT, chemotherapy; ER, estrogen receptor; ESCAT, ESMO Scale for Clinical Actionability of Molecular Targets; ESR1, estrogen receptor 1; ET, endocrine therapy; HER2, human epidermal growth factor receptor 2; m, mutation; MBC, metastatic breast cancer; MCBS, ESMO−Magnitude of Clinical Benefit Scale; PALB2, partner and localiser of BRCA2; PARP, poly (ADP−ribose) polymerase; PD, progressive disease; PIK3CA, phosphatidylinosi−tol−4,5-bisphosphate 3−kinase catalytic subunit alpha.

In the treatment of HER2-negative metastatic breast cancer (Figure 1), the metastatic site is important, because it can be both a detectable marker and a therapeutic target [23,24,25]. The presence or absence of estrogen and progesterone receptors (ER;PR) and HER2, mitotic activity, the site of metastases, and cancer recurrence, determines the treatment for metastatic breast cancer [25,26]. According to recent guidelines, endocrine therapy should be the treatment of choice for most patients with advanced or metastatic ER+, PR+/HER2 (−) breast cancer unless there is evidence of rapidly progressive visceral disease with organ dysfunction or imminent organ failure [27,28]. The association of targeted therapy with endocrine therapy has shown an improved overall survival compared to endocrine therapy alone [29,30]. The use of poly (ADP-ribose) polymerase (PARP) inhibitors as monotherapy has shown significant improvements in the PFS (progression-free survival) compared to chemotherapy in metastatic HER2-mutated germline BRCA breast cancer [31,32]. Olaparib is a selective inhibitor of the poly (ADP-ribose) polymerase (PARP) enzymes (PARP1 and PARP2), which works by taking advantage of the defect in DNA repair in cancer cells with BRCA mutations and inducing cell death [33]. In breast cancer, it is used as an adjuvant treatment for HER2-negative neoplasia, as well as in the triple negative intrinsic subtype [34,35]. Alpelisib is an inhibitor of phosphatidylinozytol 3-kinase (PI3K) with strong anti-tumor activity [36,37]. In addition to Fulvestrant, it is indicated for the treatment of women with postmenopausal breast neoplasm and men with advanced or metastatic breast cancer with positive hormone receptors (ER+, PR+), negative HER2, and mutated-PIK3CA [38,39].

In our Romanian National Health Care Program PN 3, PIK3CA mutation therapy is not settled, while testing and treatment are received by patients who can afford it. In our clinic, only one young patient with recurrent metastatic disease benefited from self-purchased anti-PIK3CA therapy. The purpose of our retrospective observational study is to demonstrate the importance of genetic determinations in the management of patients with breast cancer. Our main objective was to assess HER2 overexpression among newly diagnosed breast carcinomas, related to clinical, diagnostic, and personalized therapeutic management according to the stage. Secondary objectives were to assess HER2 positivity related to demographic, therapeutic, and evolutionary criteria (the time interval in which relapses occurred after the first line of targeted treatment and the number of subsequent therapeutic lines). We focused on assessing the aggressive behaviors of breast cancer in HER2-positive women included in the study by assessing the DFI (disease-free interval), i.e., the onset of remission, without the occurrence of a loco-regional or remote relapse for the non-metastatic stages and the PFS (progression-free survival), respectively, regarding the appearance of new metastatic lesions or the resumption of evolution through the progression of old lesions for the metastatic stages. We limited ourselves to assess the disease-free interval (DFI) of the patients that were temporarily enrolled for a period of our study, associated with successive anti-HER2 therapeutic lines administered in accordance with the existing approvals in the Romanian National Health Program PN3. In this paper, we focused on the study of drug combinations in oncology patients diagnosed with breast cancer and treated at the Sibiu Emergency County Clinical Hospital and the therapeutic impact on the PFS and DFI. The combination of Pertuzumab + Trastuzumab was a major breakthrough in the management of HER2-positive breast cancer patients.

## 2. Materials and Methods

### 2.1. Patient Selection

The patient selection in this study included all patients who presented a complete medical history since the diagnosis of breast cancer. The following were investigated for each patient included in the study:

- Histopathological and immunohistochemical results, as well as immunofluorescence tests for HER2 equivocal tissue samples collected by surgical biopsies (incisional or excisional) or ultrasound-guided biopsies using breast biopsy needles;
- BRCA tests or multigene tests on serum samples from patients who have funded themselves financially (the tests not being settled by the Romanian National Health Program), an insignificant number within the selected group of patients;
- The patient’s electronic observation sheet (the ATLAS computer program of the Sibiu County Emergency Clinical Hospital);
- Paper-based medical records (inpatient care and day care observation sheets from the hospital’s archive);
- Onc2 record sheets from the outpatient/Oncology department of the Sibiu County Emergency Clinical Hospital).

### 2.2. Study Design

This retrospective study was conducted over a 3-year period (January 2021–December 2023) at the Sibiu County Emergency Clinical Hospital. A total of 237 patients with breast carcinoma were diagnosed and admitted to the Oncology department, of which 45 had histopathologically confirmed breast cancer with HER2 overexpression through the mutational process of the EGFR-ERBB2 receptor.

Informed consent was obtained from all subjects involved in the study, and the study was conducted in accordance with the Declaration of Helsinki and by the Ethics Committee of the Sibiu County Emergency Clinical Hospital (approval no. 4952; Sibiu, Romania). This study focused on genetic determinations in the management of patients with breast cancer, specifically assessing HER2 overexpression among newly diagnosed breast neoplasias.

The study was limited in determining genetic susceptibility of patients included in the study by mutations in BRCA 1/2 repairing genes or other genes determined by multiple panels, considering financial constraints. The main objective was to assess HER2 overexpression related to clinical, diagnostic, and personalized therapeutic management according to the stage of breast cancer.

### 2.3. Inclusion and Exclusion Criteria

- Inclusion Criteria
  ○ Patients with suspected breast tumors assessed through clinical examination and mammogram.
  ○ Patients with histopathologically confirmed breast cancer showing HER2 overexpression.
  ○ Patients who provided informed consent and were included in the study conducted at the Sibiu County Emergency Clinical Hospital.
- Exclusion Criteria
  ○ Patients with negative HER2 status (0, 1+).
  ○ Patients with HER2 equivocal (2+) status/FISH- or DISH-negative.
  ○ Patients not meeting the criteria for genetic susceptibility testing due to financial limitations.

### 2.4. Follow-Up Details

Each patient included in this study was followed-up with every 6 months, over a period of 3 years, until December 2023. The evaluation was carried out both clinically and through imaging to assess the therapeutic response and the disease-free interval (DFI). For the imaging evaluation, magnetic resonance imaging (MRI), Computerized Tomography (CT) and Positron Emission Tomography-Computed Tomography scans (PET-CT) were included.

### 2.5. Immunohistochemical Detection of Hormone Receptors KI67 and HER2

Immunohistochemical staining for ER, PR, HER2, and KI67 was performed on sections of formalin-fixed paraffin-embedded breast tissue from core biopsies and subsequent surgical specimens. The thickness of breast tissue sections was 3 µm. After heating in the drying oven, the sections were stained using the Ventana BenchMark ^®^GX in automatic mode (Ventana Medical Systems Inc., Tucson, AZ, USA) for the assessment of ER, PR, HER2, and KI67. The assessment of ER and PR was based on the staining intensity (weak, moderate, intense) and the percentage of tumor cells showing nuclear immunostaining for ER and PR with a range between 0 and 100%. Breast tissue sections were considered positive for ER and PR if >1% of tumor cells showed positive nuclear staining [40]. KI67 was expressed as the percentage of the number of immunostained nuclei among the total number of nuclei of tumor cells. The evaluation criteria of HER2 status were based on the intensity of cell membrane immunostaining and the percentage of membrane-positive cells by using a score range from 0 to 3+ [13]. HER2 negativity (score 0 or 1+) was concluded when no staining or membrane staining in <10% of tumor cells or weakly partial membrane staining in more than 10% of tumor cells was observed. HER2 equivocal status (score 2+) was concluded when weak to moderate complete membrane staining in >10% of tumor cells and HER2-positive status (score 3+) was concluded when strong complete membrane staining in more than 10% of tumor cells was observed [13,41].

After performing the immunohistochemistry and immunofluorescence tests, 45 HER2-positive patients (n = 45) were identified, who were evaluated demographically and therapeutically (anti-HER2 treatment alone, double therapy, first-line, and post-progression of the disease) in all stages (I–IV). In patients whose result showed HER2 equivocal status (2+), the tissue samples were sent to an accredited genetics laboratory in the country, where immunofluorescence tests (FISH/DISH) were performed. By this method, the amplification/non-amplification of the gene encoding the HER2 protein was established.

The 2 multigene panel tests, for the patients who accepted and could financially afford to perform them, were collected in the Oncology department along with signed informed consent and sent to the accredited genetics laboratories in the country. The tests were performed with a new generation sequencing method based on the PCR reaction. In addition, 4 cases of BRCA mutations in young patients were also identified. Another 9 tests were performed only for the identification of BRCA mutations. This was the choice of each patient who preferred to know the mutant or wild-type status of these genes with prophylactic therapeutic utility for bilateralization and ovarian neoplasia.

### 2.6. Statistical Analysis

Data were statistically analyzed using the Microsoft Office Excel application (Microsoft Corp., Redmond, DC, USA) and SPSS Statistics 23.0 software (SPSS, Inc., Chicago, IL, USA). For the statistical analysis performed, two qualitative variables were used to compare the association table (Crosstabs). A significance level (*p*) of the Likelihood ratio test of *p* < 0.05 was considered statistically significant.

### 2.7. Characteristics of the Group

Demographic characteristics, hormonal status, comorbidities, and family history of breast cancer of the patients included in the study group are described in Table 1. Forty-four (44) female patients and one HER2-positive male patient with a median age of 58 years (32–73 years) were included in our study. Regarding the environment of origin, it is observed that the majority of patients were from an urban environment (34 vs. 11 cases) compared to a rural one. The hormonal status of the patients included in the study group was balanced, 21 patients being identified in the premenopausal stage (47%) and 24 in the menopausal stage (53%). From the total number of patients included in the study group, 53% (24 cases) presented comorbidities, namely, hypertension and type II diabetes, at the time of diagnosis, while 21 of them did not present any comorbidities.

## 3. Results

Although the current study is based on a group of only 45 patients, it highlighted important characteristics in patients with HER2-positive breast cancer. Main clinical features of HER2-positive breast cancer patients and those with a family history of cancer are described in Table 2. Analyzing the presence of right versus left tumors, we note the more frequent location in the right breast in 27 cases (60%), compared to 18 cases in the left mammary gland (40%). In most cases (82%), the tumors were singular, followed by six cases with bifocal tumors (14%) and only two cases of multifocal tumors (4%). Regarding the site of the breast tumors, the most frequent location was in the outer-upper quadrant (42%), followed by the inner-upper quadrant (18%) and the union of the upper quadrants (18%). Four patients had a tumor located in the central quadrant (9%) and three patients had a tumor in the inner lower quadrant (6%). In addition, three cases with occult breast carcinoma were identified (axillary adenopathy confirmed histologically and immunohistochemically, without clinically detectable mammary tumor, respectively, or imaging).

The immunohistochemical determination of hormone receptors (Table 2) revealed positivity at different degrees of estrogen receptors (ERs) and progesterone receptors (PRs). Most of the HER2-positive patients had both positive hormone receptors (58%), followed by patients with both negative hormone receptors (35%). Only 7% of the patients (three cases) had positive estrogen receptors associated with negative progesterone receptors.

Regarding the immunohistochemical analysis related to the relevant breast, the HER2-positive patients with negative estrogenic receptors and negative progesterone receptors (ER−, PR−) are associated with right breast location (*p* < 0.05).

The histopathological and immunohistochemical characteristics of the HER2-positive patients are described in Table 3. The histopathological subtype NST (no special type) or invasive ductal carcinoma predominated in 41 cases (91%), compared to lobular carcinoma, which was identified in only 3 cases (7%). A single case (2%) of metaplastic carcinoma with squamous differentiation was detected (*p* = 0.633).

The HER2 status of the patients included in our study was immunohistochemically strongly positive in a percentage of 89% (40 cases), the remaining 11%, equating to five patients, displayed amplification in the immunofluorescence tests, being initially identified with equivocal HER2 (2+).

The value of the KI67 proliferation index was 82%. An increased value of this index corresponds to a higher tumor aggressiveness. Thus, from all the patients included in the study, 17 patients (38%) presented KI67 between 50 and 90%, 20 patients (44%) between 30 and 50% and only 8 cases (18%) had KI67 reduced by below 30%.

We decided to analyze the value of KI67 in relation to hormone receptors. By using the Likelihood ratio test, a highly significant association was found between patients with RE+ RE− and a KI67 index < 30% (*p* = 0.001).

In terms of tumor grading, 38% (17 cases) had poorly differentiated tumors (G3), 27 patients (60%) had tumors with intermediate grading (G2), and only 1 patient (2%) had well−differentiated tumors (*p* = 0.649). These data correlate with the value of the proliferation index presented above and by the predominance of G2+G3 (98%). The KI67 index was 82%, with over 30% of values considered high, There is a percentage difference of mathematical association between the KI67 value and G2 + G3 of about 16%, which can be explained by the lack of a gold standard for assessing the percentage tumor aggression in terms of the KI67 value. Regarding the classification of intrinsic HER2-positive subtypes, 31 patients (69%) were identified with LUMINAL B-HER2-positive tumors (positive estrogenic hormone receptors, KI67 over 20%), and 14 patients (31%) displayed enriched HER2 tumors (hormone receptor-negative). The intrinsic subtype Luminal B HER2-positive refers to a malignant phenotype characterized by positive estrogenic receptors (ERs), positive or negative progesterone receptors (PRs), a KI67 value over 30%, and HER2 positivity. The enriched HER2 subtype is immunohistochemically characterized by the negativity of hormonal receptors and HER2 positivity.

We decided to analyze patients with HER2-positive breast cancer according to the stage of the disease (Table 1). Nine patients (20%) were diagnosed as HER2-positive in stage IV, only two in stage I (4%), fourteen cases in stage II (31%), and twenty cases in stage III (45%). The data presented show that the majority of the patients (65%) had advanced loco-regional and metastatic disease at diagnosis.

The patients included in the study group were analyzed according to the cTNM staging system (Table 1), which revealed important features. The prevalence of T2 tumors was 40% (19 cases), being the most frequent, followed by 11 cases with T3 tumors (25%) and 10 patients (24%) with T4 tumors (T4b and T4c). Only five patients (11%) presented small tumors classified as T1. The T category (tumor size) was established by clinical ruler measurement of the palpable dimensions of two perpendicular diameters, taking into account the maximum diameter. Statistical analysis does not show an association between HER2 overexpression and tumor size (*p* = 0.053 in Likelihood ratio test).

On clinical palpation of the peripheral lymph nodes, 22 cases (49%) were identified in the N1 stage and 13 patients (29%) in the N2 stage. Two cases (4%) were categorized as N3 and only 18% (eight cases) had clinically negative axilla (*p* = 0,173). The clinical status N (node) was established by palpation, without being histopathologically confirmed by biopsy. Currently, there is a reluctance of some surgeons to perform a biopsy (post-diagnosis) on any palpable peripheral adenopathy, a very important diagnostic element in achieving correct staging.

Regarding the presence of metastases (M), 36 of the HER2-positive patients (80%) did not have metastatic disease at diagnosis after performing imaging investigations (abdominal–pelvic CT scan, bone scintigraphy as appropriate, brain or bone MRI). A proportion of 20% of patients had distant metastases.

Neoadjuvant anti-HER2 treatment in combination with sequential chemotherapy (Table 4) was administered to 32 HER2-positive patients (71%) as a double blockade of anti-EGFR monoclonal antibodies (Pertuzumab + Tratuzumab), and to 2 patients (4%), following monotherapy with Trastuzumab. Of the total number of patients included in the study, 11 (25%) did not benefit from neoadjuvant anti-HER2 therapy for various reasons (patient preference, metastatic stage, per primam surgery with presentation to the Oncological Commission after surgery, cardiovascular comorbidities that risked decompensation by administering the anti-HER2 therapy).

After the end of the neoadjuvant treatment, as well as the clinical and imaging evaluation of the therapeutic response, the patients were directed to the surgical sequence. A total of 28 patients (62%) underwent radical surgery, performing radical Madden mastectomy with axillary lymph node dissection, and 16% (7 cases) benefited from conservative surgery (sectorectomy with axillary lymph node dissection). Mandatory postoperative adjuvant radiotherapy for conservative surgery and residual tumor from the breast and/or lymphatic (axillary) nodes was indicated in 31 cases, representing a rate of 69%. In one case with the identified BRCA mutation, bilateral mastectomy with homolateral axillary lymph node dissection was performed followed by subsequent breast reconstruction, which was performed in a plastic surgery center in the country. This case benefited from informed consent and prophylactic oophorectomy. In the Sibiu County Emergency Clinical Hospital, at the time of performing the mentioned surgical sequences, the sentinel lymph node technique was not possible.

The post-surgical histopathological examination (Table 5) revealed complete pathological responses (pCRs) in 19 patients (56%), without residual tumor or microscopic lymphatic invasion (yT0N0M0) and in a percentage of 44% (15 cases), pathological responses with residual disease either at the tumor level or lymphatic or both variants.

According to the postoperative therapeutic guidelines, the first anti-HER2 treatment line was performed according to the postoperative histopathological response and the presence of axillary adenopathy (cN+) at diagnosis. Twelve cases (34%) received monotherapy with TDM-1 for residual disease (ypT ≠ ypT0 +/− ypN ≠ ypN0 + M0). A proportion of 44% (20 cases) of HER2-positive patients who were treated with neoadjuvant or adjuvant anti-HER2 therapy relapsed after first-line, while 56% had complete remission or stable disease (without progression). The disease-free interval (DFI) or the time period in which we recorded a stable disease at most, in other words without progression, was recorded in 41 patients, representing 91% of the study group. The remaining four patients did not reach DFI, still having disease progression despite anti-HER2 therapy. For progressive disease (single or repeated progression), follow-up lines of anti-HER2 treatment have been established. A total of 14 patients (31%) followed two lines of anti-HER2 drugs, while for 10 patients (22%), a third line of therapy was necessary.

The types of tumor relapse were either bilateralization (1 case), or remote, as single metastasis (7 cases) or multiple metastases in different sites (12). Out of the total number of patients who presented relapse in the form of metastases, the majority of the cases presented liver and bone metastases each at a percentage of 61%, followed by regional lymph node metastases in 45% of cases, lungs and brain metastases were detected in 22%, while skin and retroorbital metastases were the rarest, being identified in one patient each (5%). The BRCA status was determined in a small number of cases (13 patients) given the financial aspects represented by the high cost of BRCA or multigene testing. Four BRCA mutations were identified in young patients, less than 45 years of age, and in nine other cases, the wild-type (non-mutant) profile was determined. In a number of 32 patients, the determination of mutations was not performed either because testing was no longer recommended at their age, or because of financial inconveniences.

## 4. Discussion

Out of 237 newly diagnosed breast cancer cases during the period January 2021–December 2023 in our clinic, only 45 (19%) were HER2-positive tumors and were therefore included in our research. Most of them, 36 cases (80%), were in the early stages, and benefited from neoadjuvant management with the anti-HER2 standard of care plus chemotherapy. pCR was performed in 19 patients (56%), who continued the same anti-HER2 treatment in the adjuvant setting as well. The remaining 15 cases had residual tumor burden and received, therefore, adjuvant second-line anti-HER2 therapy. On the other hand, 9 patients were diagnosed at stage IV and benefited from several anti-HER2 treatment lines depending on the PFS. Overall, the DFI was reached in a significant number of patients, namely, 91% (41 out of 45 cases).

The multimodal treatment of breast cancer has made substantial progress in recent years [42,43]. The involvement of modern oncology and surgical treatment options have led to a substantial benefit to patients, defining the multidisciplinary treatment of breast cancer [42,43]. The introduction of immunohistochemical testing and genetic screening has led to the prioritization of therapy and a correct approach to initiating treatment [42,44].

The difference in the HER2 positivity rate of 19% for the 237 new cases diagnosed at the Sibiu County Emergency Clinical Hospital, compared to the study of Rüschoff J. et al., is probably due to the small group of patients included in our retrospective study, compared to the 15,992 histopathological samples collected from 160 oncological centers in Germany [45]. However, the 19% obtained in our group of patients diagnosed with breast cancer falls within the range mentioned also by international studies and guidelines [46,47,48].

The histopathological gradings G2 and G3 and the KI67 proliferation index, both variables characterizing the malignant phenotype specific to the tumor, correlate with aggressive evolution, translating into the increased risk of loco-regional and remote relapse.

An unfavorable influence on the evolution of the disease is the stage of the disease and the lymph node status (palpable) at diagnosis; 82% of the HER2-positive patients had cN+. The HER2-positive patients mainly were at advanced stages at presentation (III and IV), equating to 65% of all the women included in our study group. A proportion of 11% were identified as having a family history of breast cancer, which correlates with a high susceptibility to breast neoplasia.

HER2-positive breast cancer has an unfavorable prognosis, with an amplified risk of relapse and a more aggressive course of the disease, which is shown by the 44% of relapses after the first line of treatment with monoclonal antibodies.

The percentage of complete post-neoadjuvant pathological responses (targeted anti- HER2 combination therapy and sequential chemotherapy) reveals the effectiveness of the targeted treatment. The discovery and implementation of the National Health Program for new molecules directed against HER2 has allowed for the administration of follow-up lines of targeted treatment so that the overall survival is prolonged. A total of 14 patients benefited from two lines of therapy, and 10 patients received three lines of anti-HER2 therapy.

The most widely used anti-HER2 treatment was double blockade with Pertuzumab and Trastuzumab both as a neoadjuvant (71%) and adjuvant (58%) as well as in the first line of therapy of metastatic disease (100%). The double blockade confers superior therapeutic efficiency by blocking two loci of the EGFR/ERBB 2 receptor.

Our retrospective study, conducted over a limited period of time, did not allow for the correlation of the tumor aggressiveness conferred by HER2 overexpression with the status of the hormone receptors. The patients underwent radical surgical treatment (mastectomy) over conservative surgery (64% versus 16%), possibly due to the preferences of women with breast neoplasia and/or the surgeon. All the patients who underwent the surgical sequence benefited from axillary lymphodissection, the sentinel lymph node technique not being available in the Sibiu County Emergency Clinical Hospital during the period of our study. At the end of the three-year follow-up analysis, there were no deaths among the patients enrolled in our study. This is to highlight once again the effectiveness of treatment and a rapidly evolving era of oncology therapies aimed at increasing survival and quality of life.

More than half of the patients included in this study achieved pCR and continued with the same anti-HER2 therapy in the neoadjuvant setting. The outcomes suggest the importance of neoadjuvant therapy in prolonged DFS. Patients with HER2-positive stage IV breast cancer have benefited from several subsequent lines of anti-HER2 therapy with a prolonged PFS. It is important to emphasize once again that following the correct treatment strategies in HER2-positive patients in the neoadjuvant–surgery–adjuvant settings led to high pCR rates and favorable survival outcomes. Although the recommendations of the international guidelines were followed, the chemotherapeutic regimens were personalized, with the aim of increasing treatment compliance and maintaining the intensity of the doses.

## 5. Conclusions

The HER2 biomarker is a predictive prognostic indicator for the evolution of neo-plastic disease, which gives it an augmented aggressiveness, marked in the results of our study by the reduction in the disease-free interval (DFI) and by the occurrence of relapses or by the evolution of metastatic disease.

Anti-HER2 therapy in any therapeutic stage (neoadjuvant, adjuvant, first line in metastatic disease) has shown increased efficiency in blocking these tyrosine kinase receptors, evidenced by the high percentage of complete pathological responses, as well as the considerable percentage (47%) of complete remissions and stationary disease, in relation to the HER2-positive patient group. The errors in our observational study can derived from the short follow-up interval. This was chosen as a result of the introduction, in recent years, of double anti-HER2 blockade in the targeted therapy of these patients. Until recently, monotherapy with Trastuzumab was used. This is the reason why a correlation with the overall survival and mortality was not achieved, these variables requiring a much longer evaluation period.

BRCA testing with the aim of prophylaxis of contralateral recurrence and oophorectomy to decrease the risk of ovarian carcinoma failed for socioeconomic reasons due to the high cost of these tests, which cannot be borne by most of our patients.

Due to the short time period in which the research was conducted, aspects such as the correlation between tumor aggressiveness due to HER2 overexpression could not be studied. A detailed analysis of survival data should also be considered, becoming a goal of a future update of our research. We are very interested in the DFS rates of our HER2-positive breast cancer patients, evaluated as the time to recurrence and recurrence sites. Whether the immunohistochemical characteristics will change at relapse remains a question to be answered in the future. Every relapse should undergo biopsy whenever possible, since therapeutic strategies rely on pathological characteristics. In the future, we intend to continue the current study, being part of another prospective study that envisages a thorough follow-up of patients over a longer period of time.

## Figures and Tables

**Table 1:** Demographic characteristics and main clinical features of HER2-positive patients.

Total Number of Patients with HER2-Positive Breast Cancer (2021–2023) N * (%)	Environment of Origin in HER2-Positive Patients N (%)	Hormonal Status of HER2-Positive Patients N (%)	Comorbidities N (%)
45 (100%)	Urban 34 (76%)	Premenopause 21 (47%)	Hypertension and type II diabetes 24 (53%)
Age of patients: ≤45 years: 3 (7%) 45–73 years: 42 (93%)	Rural 11 (24%)	Menopause 24 (53%)	Absent 21 (47%)
Stage at diagnosis N (%)	Tumor size (T) N (%)	Palpable axillary lymph nodes (N) N (%)	Metastatic disease (M) N (%)
I 2 (4%)	T1 5 (11%)	N0 8 (18%)	M0 36 (80%)
II 14 (31%)	T2 19 (40%)	N1 22 (49%)	M1 9 (20%)
III 20 (45%)	T3 11 (25%)	N2 13 (29%)	
IV 9 (20%)	T4a	N3 2 (4%)	
	0 T4b 8 (20%) T4c 2 (4%)		

* N = Number of patients.

**Table 2:** Main clinical features of HER2-positive breast cancer patients.

Localization of HER2-Positive Tumors Depending on the Right/Left Breast N * (%)	The Incidence of HER2-Positive Breast Tumors Depending on the Quadrant N (%)	Types of HER2-Positive Tumors N (%)	Family History of Cancer N (%)	Positivity of ER ******* and PR ******** in HER2-Positive Patients
Right breast 27 (60%)	UOQ ** 19 (42%)	Single 37 (82%)	First-degree relatives with breast cancer	(ER+, PR+) 26 (58%)
			5 (11%)	(ER+, PR−) 3 (7%)
				(ER−, PR−) 16 (35%)
Left breast 18 (40%)	UIQ ***	Bifocal	Other neoplasia	
	8 (18%)	6 (14%)	4 (9%)	
	UUQ **** 8 (18%)	Multifocal 2 (4%)		
	ILQ *****			
	3 (6%)			
	CQ ****** 4 (9%)			
	Occult (Tx) 3 (7%)			

* N = number of patients. ** UOQ = upper-outer quadrant. *** UIQ = upper-inner quadrant. **** UUQ = union of the upper quadrant. ***** ILQ = inner-lower quadrant. ****** CQ = central quad-rant, ******* ER = estrogen receptor; ******** PR = progesterone receptor.

**Table 3:** Histopathological and immunohistochemical characteristics of HER2-positive patients.

Histopathological Type of Breast Cancer N * (%)	HER2 Overexpression N (%)	KI67 N (%)	Tumor Grade N (%)
Ductal invasive carcinoma (NST **) 41 (91%)	HER2-positive (3+) for IHC *** 40 (89%)	<30% 8 (18%)	G1 1 (2%)
Invasive lobular carcinoma 3 (7%)	HER2-equivocal (2+)	30–50% 20 (44%)	G2 27 (60%)
	5 (11%)		
Metaplastic carcinoma with squamous differentiation 1 (2%)		50–90% 17 (38%)	G3 17 (38%)

* N = number of patients; ** NST = no special type; *** IHC = immunohistochemistry.

**Table 4:** Oncological treatment of HER2-positive patients.

Anti-HER2-NaT + ChT ******* N * (%)	Anti-HER2-AT *****, 1st Line N (%)	Surgery N (%)	RT *** N (%)
(No) NaT **** 11 (25%)	Trastuzumab alone 3 (8%)	RM with ALND ****** 28 (62%)	No RT 14 (31%)
Double anti-HER2 blockade 32 (71%)	TDM-1 ** alone	RM ******** with subsequent reconstruction and prophylactic oophorectomy	Adjuvant RT 31 (69%)
	12 (34%)	1 (2%)	
Anti-HER2 monotherapy 2 (4%)	Double anti-HER2 blockade 21 (58%)	Sectorectomy with ALND 7 (16%)	
		No surgery 9 (20%)	

* N = number of patients; ** TDM-1 = Trastuzumab emtansine; *** RT = radiation therapy; **** NaT = neoadjuvant treatment; ***** AT = adjuvant treatment; ****** ALND = axillary lymph node dissection; ******* ChT = chemotherapy; ******** RM = radical mastectomy.

**Table 5:** The relationship between anti-HER2 positivity and the evolution of breast cancer.

The Occurrence of Relapse after 1st Anti-HER2 Therapeutic Line N * (%)	Disease-Free Interval (DFI) N (%)	Anti-HER2 Treatment Lines N (%)	Histopathological Response after Neoadjuvant Anti-HER2 Therapy N (%)
With relapse 20 (44%)	Without DFI 4 (9%)	Single treatment line 21 (47%)	pCR **
			19 (56%)
No relapse 25 (56%)	With DFI	Two lines of treatment	Partial response
	41 (91%)	14 (31%)	15 (44%)
		Three lines of treatment 10 (22%)	

* N = number of patients; ** pCR = pathological complete response.

## Data Availability

The datasets used and/or analyzed during the current study are available from the corresponding author on reasonable request.
